# Disambiguation of biomedical text using diverse sources of information

**DOI:** 10.1186/1471-2105-9-S11-S7

**Published:** 2008-11-19

**Authors:** Mark Stevenson, Yikun Guo, Robert Gaizauskas, David Martinez

**Affiliations:** 1 Department of Computer Science, University of Sheffield, Regent Court, 211 Portobello, Sheffield, S1 4DP, UK; 2 NICTA Victoria and Department of Computer Science, Software Engineering, University of Melbourne, Victoria 3010, Australia

## Abstract

**Background:**

Like text in other domains, biomedical documents contain a range of terms with more than one possible meaning. These ambiguities form a significant obstacle to the automatic processing of biomedical texts. Previous approaches to resolving this problem have made use of various sources of information including linguistic features of the context in which the ambiguous term is used and domain-specific resources, such as UMLS.

**Materials and methods:**

We compare various sources of information including ones which have been previously used and a novel one: MeSH terms. Evaluation is carried out using a standard test set (the NLM-WSD corpus).

**Results:**

The best performance is obtained using a combination of linguistic features and MeSH terms. Performance of our system exceeds previously published results for systems evaluated using the same data set.

**Conclusion:**

Disambiguation of biomedical terms benefits from the use of information from a variety of sources. In particular, MeSH terms have proved to be useful and should be used if available.

## Background

The number of documents discussing biomedical science is growing at an ever increasing rate, making it difficult to keep track of recent developments. Automated methods for cataloging, searching and navigating these documents would be of great benefit to researchers working in this area, as well as having potential benefits to medicine and other branches of science. Lexical ambiguity, the linguistic phenomenon where a term (word or phrase) has more than one potential meaning, makes the automatic processing of text difficult. For example, "cold" has several possible meanings in the Unified Medical Language System (UMLS) Metathesaurus [1] including "common cold", "cold sensation" and "Chronic Obstructive Airway Disease (COLD)". Weeber *et al. *[2] analysed MEDLINE abstracts and found that 11.7% of phrases were ambiguous relative to the UMLS Metathesaurus.

The ability to accurately identify the meanings of terms is an important step in automatic text processing. It is necessary for applications such as information extraction and text mining which are important in the biomedical domain for tasks such as automated knowledge discovery. The NLM Indexing Initiative [3] attempted to automatically index biomedical journals with concepts from the UMLS Metathesaurus and concluded that lexical ambiguity was the biggest challenge in the automation of the indexing process. Friedman [4] reported that an information extraction system originally designed to process radiology reports had problems with ambiguity when it was applied to more general biomedical texts. During the development of an automated knowledge discovery system Weeber *et al. *[5] found that is was necessary to resolve the ambiguity in the abbreviation MG (which can mean 'magnesium' or 'milligram') in order to replicate a well-known literature-based discovery concerning the role of magnesium deficiency in migraine headaches [6].

Word Sense Disambiguation (WSD) is the process of resolving lexical ambiguities. WSD has been actively researched since the 1950s and is regarded as an important part of the process of understanding natural language texts. A comprehensive description of current work in WSD is beyond the scope of this paper although overviews may be found in [7,8]. Schuemie *et al. *[9] provide an overview of WSD in the biomedical domain. Previous researchers have used a variety of approaches for WSD of biomedical text. Some of them have taken techniques proven to be effective for WSD of general text and applied them to ambiguities in the biomedical domain, while others have created systems using domain-specific biomedical resources. However, there has been no direct comparison of which information sources are the most useful or whether combining a variety sources, a strategy which has been shown to be successful for WSD in the general domain [10,11], also improves results in the biomedical domain.

This paper compares the effectiveness of a variety of information sources for WSD in the biomedical domain. These include features which have been commonly used for WSD of general text as well as information derived from domain-specific resources, including MeSH terms.

The remainder of this section provides an overview of various approaches to WSD in the biomedical domain. The Methods section outlines our approach, paying particular attention to the various types of information used by our system. An evaluation of this system is presented in the Results section, the implications of which can be found in the Discussion section.

### The NLM-WSD data set

Research on WSD for general text in the last decade has been driven by the SemEval frameworks  which provide a set of standard materials for a variety of semantic evaluation tasks [12]. At this point there is no specific collection for the biomedical domain in SemEval, but a test collection for WSD in biomedicine, the NLM-WSD data set [2], is used as a benchmark by many independent groups. (An alternative collection is described by Widdows *et al. *[13], although the authors acknowledge that the low levels of inter-annotator agreement for the sense tags make the use of this data problematic.) The Unified Medical Language System (UMLS) Metathesaurus was used to define the set of possible meanings in the NLM-WSD data set. In UMLS strings are mapped onto concepts, indicating their meaning. Strings which map onto more than one concept are ambiguous. For example, the string "culture" maps onto the concepts 'Anthropological Culture' (e.g. "The aim of this paper is to describe the origins, initial steps and strategy, current progress and main accomplishments of introducing a quality management *culture *within the healthcare system in Poland.") and 'Laboratory Culture' (e.g. "In peripheral blood mononuclear cell *culture *streptococcal erythrogenic toxins are able to stimulate tryptophan degradation in humans"). 50 terms which are ambiguous in UMLS and occur frequently in MEDLINE were chosen for the NLM-WSD data set. 100 instances of each term were selected from citations added to the MEDLINE database in 1998 and manually disambiguated by 11 annotators. Twelve terms were flagged as "problematic" due to substantial disagreement between the annotators. There are an average of 2.64 possible meanings per ambiguous term and the most ambiguous term, "cold", has five possible meanings. Concepts which were judged to be very similar in meaning were merged. For example, two concepts for "depression": 'Depressive episode, unspecified' and 'Mental Depression'. In addition to the meanings defined in UMLS, annotators had the option of assigning a special tag ("none") when none of the meanings in UMLS were judged to be appropriate.

**Figure 1:**
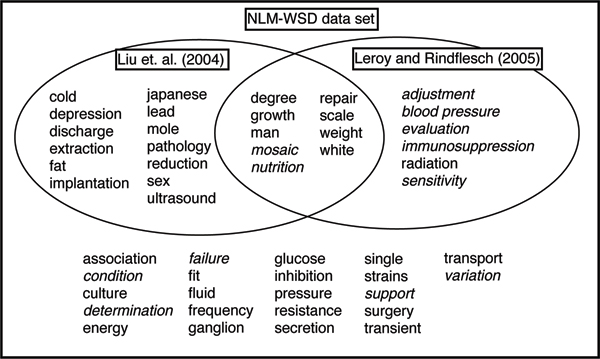
**The NLM-WSD test set and some of its subsets**. The 12 terms which Weeber *et al. *[2] described as "problematic" due to low levels of agreement between annotators are shown in italics. The test set used by Joshi *et al. *[16] comprises the set union of the terms used by Liu *et al. *[14] and Leroy and Rindflesch [15] while the "common subset" is formed from their intersection.

Various researchers have chosen to evaluate their systems against subsets of this data set. Liu *et al. *[14] used a set of 22 terms, saying "We excluded 12 [terms] that Weeber et al. considered problematic, as well as 16 terms in which the majority sense occurred with over 90% of instances." However, the 22 terms used to evaluate their system include "mosaic" and "nutrition" which Weeber *et al. *[2] flagged as problematic. Leroy and Rindflesch [15] used a set of 15 terms for which the majority sense accounted for less than 65% of the instances. Joshi *et al. *[16] evaluated against the set union of those two sets, providing 28 ambiguous terms. McInnes *et al. *[17] used the set intersection of the two sets (dubbed the "common subset") which contained 9 terms. The terms that form these various subsets are shown in Figure 1.

The 50 terms which form the NLM-WSD data set represent a range of challenges for WSD systems. The Most Frequent Sense (MFS) heuristic has become a standard baseline in WSD [18] and is simply the accuracy which would be obtained by assigning the most common meaning of a term to all of its instances in a corpus. Despite its simplicity, the MFS heuristic is a hard baseline to beat, particularly for unsupervised systems, because it uses hand-tagged data to determine which sense is the most frequent. Analysis of the NLM-WSD data set showed that the MFS over all 50 ambiguous terms is 78%. The different subsets have lower MFS, indicating that the terms they contain are more difficult to disambiguate. The 22 terms used by Liu *et al. *[14] have an MFS of 69.9% while the set used by Leroy and Rindflesch [15] has an MFS of 55.3%. The union and intersection of these sets have MFS of 66.9% and 54.9% respectively.

### WSD of biomedical text

A standard approach to WSD is to make use of supervised machine learning systems which are trained on examples of ambiguous words in context along with the correct sense for that usage. The models created are then applied to new examples of that word to determine the sense being used.

Approaches which are adapted from WSD of general text include [14]. Their technique uses a supervised learning algorithm with a variety of features consisting of a range of collocations of the ambiguous word and all words in the abstract. They compared different supervised machine learning algorithms and found that a decision list worked best. Their best system correctly disambiguated 78% of the occurrences of 22 ambiguous terms in the NLM-WSD data set (see Figure 1).

Joshi *et al. *[16] also use collocations as features and experimented with five supervised learning algorithms: Support Vector Machines, Naive Bayes, decision trees, decision lists and boosting. The Support Vector Machine performed best scoring 82.5% on a set of 28 words (see Figure 1) and 84.9% on the 22 terms used by Liu *et al. *[14]. Performance of the Naive Bayes classifier was comparable to the Support Vector Machine, while the other algorithms were hampered by the large number of features.

Examples of approaches which have made use of knowledge sources specific to the biomedical domain include Leroy and Rindflesch [15] who used information from the UMLS Metathesaurus. They used the MetaMap tool [19] which identifies the relevant UMLS concepts for a piece of text. Leroy and Rindflesch used knowledge about whether the ambiguous word is the head word of a phrase identified by MetaMap, the ambiguous word's part of speech, semantic relations between the ambiguous words and surrounding words from UMLS as well as semantic types of the ambiguous word and surrounding words. Naive Bayes was used as a learning algorithm. This approach correctly disambiguated 65.5% of word instances from a set of 15 terms (see Figure 1). Humphrey *et al. *[20] presented an unsupervised system that also used semantic types from UMLS. They constructed semantic type vectors for each word from a large collection of MEDLINE abstracts. This allowed their method to perform disambiguation at a coarser level, without the need for labeled training examples. In most cases the semantic types can be mapped to the UMLS concepts used to annotate instances in the NLM-WSD corpus but not for all terms. In addition, this approach could not disambiguate instances which had been annotated with the "none" tag which indicated that none of the meanings in UMLS were judged to be appropriate. Five terms were excluded from their evaluation, four ("cold", "man", "sex" and "weight") because the semantic types could not be mapped onto UMLS concepts and the other ("association") because all instances of that term were assigned the "none" tag. In addition, only 67% of the instances for the remaining 45 terms were used for evaluation and, since instances with the "none" tag were also excluded, their system was only evaluated against an average of 54% of the instances of these terms. An accuracy of 78.6% was reported across these instances. McInnes *et al. *[17] also made use of information provided by MetaMap. In UMLS each concept has a Concept Unique Identifier (CUI) and these are also assigned by MetaMap. The information contained in CUIs is more specific than in the semantic types applied by Leroy and Rindflesch [15] and Humphrey *et al. *[20]. For example, two of the CUIs for the term "cold" in UMLS, "C0205939: Common Cold" and "C0024117: Chronic Obstructive Airway Disease", share the same semantic type: "Disease or Syndrome". McInnes *et al. *[17] were interested in exploring whether the more specific information contained in CUIs was more effective than UMLS semantic types. Their best result was reported for a system which represented each sense by all CUIs which occurred at least twice in the abstract surrounding the ambiguous word. They used a Naive Bayes classifier as the learning algorithm and reported an accuracy of 74.5% on the set of ambiguous terms tested by Leroy and Rindflesch [15] and 80.0% on the set used by Joshi *et al. *[16]. They concluded that CUIs are more useful for WSD than UMLS semantic types but that they are not as robust as features which are known to work in general English, such as unigrams and bigrams. Unfortunately, direct comparison of the various WSD systems which have been evaluated on the NLM-WSD data set is not straightforward. Firstly, as we have described, systems have been tested against a variety of ambiguous terms. A more subtle problem arises in the way in which researchers have chosen to present their results. With the exception of unsupervised systems [15,20], which do not require training data, all approaches involve training a classifier using some portion of the available data and then testing against the remaining unseen portion. These supervised approaches normally involve choices over how to set the parameters which define the group of features used. For example, Liu *et al. *[14] compared a total of 22 different feature sets by varying the size of the context window around the ambiguous word and the terms which are extracted. One approach [14,16] is to experiment with a variety of parameters and choose the best one for each ambiguous term. For example, the 78% accuracy figure quoted by Liu *et al. *[14] is obtained by choosing the result from the best classifier for each of the 22 terms used in their evaluation. We refer to this as *per-term parameter setting*. An alternative methodology involves applying the same parameters to all terms. For example, the results reported by McInnes *et al. *[17] are obtained by using the same parameters for all terms rather than selecting the best result for each. We call this *global parameter setting*.

It would be preferable to automate the process of parameter setting as far as possible however this would be difficult for per-term parameter setting, particularly for a data set such as NLM-WSD where there are only 100 instances for each ambiguous term and many senses with occur only a few times. The alternative approach, global parameter setting, is less affected by this problem and has the advantage that the settings are more likely to be suitable for terms other than the ones which are contained in the test collection. The global parameter setting methodology is used in the experiments described later in this paper.

## Methods

Our approach is to adapt a state-of-the-art WSD system to the biomedical domain by augmenting it with additional domain-specific and domain-independent information sources. Our basic system [21] participated in the Senseval-3 challenge [22] with a performance close to the best system for both the English and Basque lexical sample tasks. The method is based on a supervised learning approach and uses features derived from text around the ambiguous word which are domain independent. We refer to these as *linguistic *features. This feature set has been adapted for the disambiguation of biomedical text by adding further linguistic features and two different types of domain-specific features: CUIs (as used by McInnes *et al. *[17]) and Medical Subject Heading (MeSH) terms.

### Features

Our feature set contains a number of parameters which were set empirically (e.g. threshold for unigram frequency in the linguistic features). In addition, we use the entire abstract as the context of the ambiguous term for relevant features rather than just the sentence containing the term. Effects of varying these parameters are similar to results reported in previous work [14,16,17], for example using the entire abstract as context yields more accurate results than using only the sentence containing the ambiguous term. Since these results are not novel we do not report them in this paper.

#### Linguistic features

The system uses a wide range of domain-independent features which are commonly used for WSD.

• Local collocations: A total of 41 features which extensively describe the context of the ambiguous word and fall into two main types: (1) bigrams and trigrams containing the ambiguous word constructed from lemmas, word forms or PoS tags (assigned using maximum-entropy-based part of speech tagger [23]) and (2) preceding/following lemma/word-form of the content words (adjective, adverb, noun and verb) in the same sentence with the target word. For example, consider the sentence below with the target word *adjustment*.

"Body surface area *adjustments *of initial heparin dosing..."

The features would include the following: left-content-word-lemma "*area adjustment*", right-function-word-lemma "*adjustment of *", left-POS "NN NNS", right-POS "NNS IN", left-content-word-form "*area adjustments*", right-function-word-form "*adjustment of *", etc.

• Salient bigrams: Salient bigrams within the abstract with high log-likelihood scores computed from the NLM-WSD corpus, as described by Pedersen [24]. In the experiments, bigrams that occur more than once and have a log-likelihood higher than 6.635 are included as features.

• Unigrams: Lemmas of all content words (nouns, verbs, adjectives, adverbs) in the target word's sentence and, as a separate feature, lemmas of all content words within a ± 4-word window around the target word, excluding those in a list of corpus-specific stopwords (e.g. "ABSTRACT", "CONCLUSION"). In addition, the lemmas of any unigrams which appear at least twice in the entire corpus and are found in the abstract are also included as features. This feature was not used by [21], but Joshi *et al. *[16] found it to be useful for this task.

A previous version of our system [25] included syntactic dependencies, such as subject and noun-modifier of ambiguous terms, as an additional feature. These features were also used by Agirre and Martinez [21] and were extracted by a set of manually-created heuristics applied to part of speech tagged text. However, we found that removing these features led to a small increase in performance. The likely reason for this is that these features are noisy since the dependencies are difficult to identify accurately. In addition the heuristics used were not developed to be applied on biomedical documents.

### Concept Unique Identifiers (CUIs)

We follow the approach presented by McInnes *et al. *[17] to generate features based on UMLS Concept Unique Identifiers (CUIs). The MetaMap program [19] identifies all words and terms in a text which could be mapped onto a UMLS CUI. MetaMap does not disambiguate the senses of the concepts; instead it enumerates all the possible combinations of the concept names found. For example, MetaMap will segment the phrase "Body surface area adjustments of initial heparin dosing ..." into two chunks: "Body surface area adjustments" and "of initial heparin dosing". The first chunk will be mapped onto four CUIs, two with the concept name "Body Surface Area": "C0005902: Diagnostic Procedure" and "C1261466: Organism Attribute" and a further pair with the name "Adjustments": "C0456081: Health Care Activity" and "C0871291: Individual Adjustment". CUIs which occur more than three times in the abstract containing the ambiguous word are included as features.

#### Medical Subject Headings (MeSH)

The final feature is also specific to the biomedical domain. Medical Subject Headings (MeSH) [26] is a controlled vocabulary for indexing biomedical and health-related information and documents. MeSH terms are manually assigned to abstracts by human indexers. The latest version of MeSH contains over 24,000 terms organised into an 11-level hierarchy. The terms assigned to the abstract in which each ambiguous word occurs are used as features. For example, the abstract containing the example phrase in the previous paragraph has been assigned 16 MeSH terms including "M01.060.116.100: Aged", "M01.060.116.100.080: Aged, 80 and over", "D27.505.954.502.119: Anticoagulants" and "G09.188.261.560.150: Blood Coagulation". To our knowledge MeSH terms have not been previously used as a feature for WSD of biomedical documents.

### Learning algorithms

We compared three machine learning algorithms which have previously been shown to be effective for WSD tasks.

The **Vector Space Model **is a memory-based learning algorithm which was used by [21]. Each occurrence of an ambiguous word is represented as a binary vector in which each position indicates the occurrence/absence of a feature. A single centroid vector is generated for each sense during training. These centroids are compared with the vectors that represent new examples using the cosine metric to compute similarity. The sense assigned to a new example is that of the closest centroid.

The **Naive Bayes **classifier is based on a probabilistic model which assumes conditional independence of features given the target classification. It calculates the posterior probability that an instance belongs to a particular class given the prior probabilities of the class and the conditional probability of each feature given the target class.

**Support Vector Machines **have been widely used in classification tasks. SVMs map feature vectors onto a high dimensional space and construct a classifier by searching for the hyperplane in that space that gives the greatest separation between the classes.

We used our own implementation of the Vector Space Model and Weka implementations [27] of the other two algorithms. A linear kernel was used for the Support Vector Machine.

## Results

This system was applied to the entire NLM-WSD data set. Experiments were carried out using each of the three types of features (linguistic, CUI and MeSH) both alone and in combination. Ten-fold cross validation was applied and the figures we report are averaged across all ten runs.

Results from this experiment are shown in Table 1, which lists the performance using combinations of learning algorithm and features. The figure shown for each configuration represents the percentage of instances of ambiguous terms which are correctly disambiguated.

**Table 1:** Results from WSD system. Results from WSD system applied to various sections of the NLM-WSD data set using a variety of features and machine learning algorithms. The best results obtained by our system are highlighted in bold font. Results from baseline and previously published approaches are included for comparison.

	Features						
Data sets	Linguistic	CUI	MeSH	CUI+MeSH	Linguistic+MeSH	Linguistic+CUI	Linguistic+MeSH+CUI

	Vector space model						

All words	87.0	85.8	81.9	86.9	**87.9**	87.3	87.5
Joshi subset	82.1	79.6	76.6	81.4	**83.3**	82.4	82.8
Leroy subset	77.5	74.4	70.4	75.8	**79.7**	78.7	78.9
Liu subset	84.0	81.3	78.3	83.4	**84.8**	83.9	84.2
Common subset	79.1	75.1	70.4	76.9	**81.1**	80.0	79.7

	Naive Bayes						

All words	86.4	81.2	85.7	81.1	86.4	81.7	81.8
Joshi subset	80.9	73.4	80.1	73.7	81.1	74.1	74.5
Leroy subset	76.9	66.1	74.6	65.9	77.5	66.5	67.2
Liu subset	82.1	75.4	81.7	75.3	82.7	76.3	76.6
Common subset	77.2	66.1	74.7	65.8	79.0	66.7	67.4

	Support Vector Machine						

All words	85.9	83.5	85.3	84.5	86.2	85.3	86.0
Joshi subset	80.1	76.4	79.5	78.0	80.9	79.1	80.3
Leroy subset	75.5	69.7	72.6	72.0	77.1	74.5	76.3
Liu subset	81.7	78.2	81.0	80.0	82.3	80.6	81.7
Common subset	76.3	69.8	71.6	73.0	78.1	75.1	76.9

		Previous Approaches					
		
		Per-term		Global			
		
	MFS baseline	Liu *et al. *(2004)	Joshi *et al. *(2005)	Leroy and Rindflesch (2005)		Joshi *et al. *(2005)	McInnes *et. al. *(2007)

All words	78.0	-	-	-		86.2	85.3
Joshi subset	66.9	-	82.5	-		80.9	80.0
Leroy subset	55.3	-	77.4	65.5		75.7	74.5
Liu subset	69.9	78.0	84.9	-		83.3	81.9
Common subset	54.9	-	79.8	68.8		78.1	75.6

The best performance is obtained using a combination of the linguistic and MeSH features, a pattern observed across all test sets and machine learning algorithms. Although the increase in performance gained from using both the linguistic and MeSH features compared to only the linguistic features is modest, it is statistically significant (Wilcoxon Signed Ranks Test, *p *< 0.05), as is the difference between using both linguistic and MeSH features compared with using the MeSH features alone (*p *< 0.01).

The Vector Space Model learning algorithm performs significantly better than both Support Vector Machines and Naive Bayes (Wilcoxon Signed Ranks Test, *p *< 0.01). This pattern is observed regardless of which set of features is used, and it is consistent with the results over SemEval data [21].

Performance using MeSH terms as the only feature is better than using CUIs alone when the Naive Bayes and Support Vector Machine Learning algorithms are used. However, this is reversed for the Vector Space Model. The most likely reason is that the MeSH terms are far more sparse than CUIs (see Discussion section) which hinders this algorithm's performance.

### Per-word analysis

Table 2 shows the results of our best performing system (combination of linguistic and MeSH features using the Vector Space Model learning algorithm). Comparable results for previous supervised systems are also reported where available. To allow direct comparison the results from Joshi *et. al. *[16] are computed using global parameter setting (see WSD of Biomedical Text section). An equivalent set of results are not available for Liu *et al. *[14]. Results from Humphrey *et al. *[20] are also omitted since their system was evaluated against only some of the instances of each term. The MFS baseline for each term is shown in the leftmost column.

**Table 2:** Per-word performance of best reported systems

	MFS baseline	Leroy and Rindflesch (2005)	Joshi et. *al *(2005)	McInnes *et al*.(2007)	Reported system
adjustment	62	57	71	70	**73**
association	100	-	**100**	97	**100**
*blood pressure*	54	46	50	46	**53**
cold	86	-	**90**	**89**	88
*condition*	90	-	**89**	89	**89**
culture	89	-	**96**	94	95
degree	63	68	89	79	**93**
depression	85	-	84	81	**86**
determination	79	-	85	81	**87**
discharge	74	-	95	**96**	94
*energy*	99	-	**99**	**99**	98
evaluation	50	57	67	73	**81**
extraction	82	-	84	**86**	85
failure	71	-	69	**73**	**73**
fat	71	-	**84**	77	**84**
fit	82	-	81	87	**88**
fluid	100	-	**100**	99	**100**
frequency	94	-	**95**	94	94
ganglion	93	-	95	94	**96**
glucose	91	-	**92**	90	91
growth	63	62	69	69	**72**
immunosuppression	59	61	79	75	**81**
implantation	81	-	**93**	92	91
inhibition	98	-	**98**	**98**	**98**
japanese	73	-	76	76	**77**
lead	71	-	88	90	**94**
man	58	80	**89**	80	86
mole	83	-	**94**	87	88
mosaic	52	66	**87**	75	85
nutrition	45	48	52	49	**57**
pathology	85	-	85	84	**86**
*pressure*	96	-	91	93	**95**
radiation	61	72	81	81	**85**
reduction	89	-	91	**92**	88
repair	52	81	87	**93**	86
resistance	97	-	**97**	96	**97**
scale	65	84	76	83	**88**
secretion	99	-	**99**	**99**	**99**
sensitivity	49	70	85	92	**93**
sex	80	-	**87**	**87**	**87**
single	99	-	**99**	98	**99**
strains	92	-	**93**	92	**93**
support	90	-	89	**91**	90
*surgery*	98	-	**98**	94	97
transient	99	-	**99**	98	**99**
transport	93	-	**94**	93	93
ultrasound	84	-	87	85	**88**
variation	80	-	88	91	**94**
weight	47	68	**83**	79	82
white	49	62	71	74	**81**

The performance of Leroy and Rindflesch's system is always lower than the best result for each word. The systems reported by Joshi *et al. *[16] and McInnes *et al. *[17] are better than, or the same as, all other systems for 23 and 11 words respectively. The system reported here achieves results equal to or better than previously reported systems for 33 terms.

There are five terms for which the performance of our approach is actually lower than the MFS baseline (shown in *italics*) in Table 2. (In fact, the baseline outperforms all systems for three of these terms.) The performance of our system is within 1% of the baseline for five of these terms. The remaining pair, "blood pressure" and "failure", are included in the set of problematic words [2]. Examination of the possible senses show that they include pairs with similar meanings. For example, the two senses which account for the majority (98%) of the instances of "blood pressure", which refer to the blood pressure within an organism and the result obtained from measuring this quantity, are very closely related semantically.

### Linguistic features

Our WSD algorithm uses a wider range of linguistic features than previous approaches. Table 3 shows a comparison of each of the three types of linguistic features described in the Features section. Each type of feature is used alone and as part of a pair. Performance of each type of feature used alone is above the relevant MFS baseline, indicating that all three provide useful information for disambiguation. Unigrams are the most effective, followed by salient bigrams with local collocations the least effective. A possible reason for this may lie in the fact that local collocations comprise an extensive feature set, some of which may be redundant or noisy. For all words the pairing of local collocations with unigrams is the most effective with performance only 0.1% less accurate than combining all three types of linguistic features. However, combining salient bigrams with unigrams generates the best results over each of the four subsets and actually outperforms the combination of all three feature types for two of them.

**Table 3:** Contribution of linguistic features. Results from various combinations of types of linguistic features, as described in Features section, combined using Vector Space Model learning algorithm. LC = Local Collocations, SB = Salient Bigrams and U = Unigrams.

	Features					
Data sets	LC	SB	U	SB+U	LC+SB	LC+U

All words	79.2	82.0	86.9	85.9	86.3	86.9
Joshi subset	72.6	74.4	81.6	82.3	81.0	82.0
Leroy subset	66.2	66.9	76.7	77.5	76.5	77.3
Liu subset	75.7	76.2	83.4	84.3	82.7	83.9
Common subset	69.6	77.7	79.3	79.1	77.6	78.8

## Discussion

Our experiment shows that each of the three types of information (linguistic, CUIs and MeSH) can be used to create a classifier which achieves a reasonable level of disambiguation, since performance exceeds the relevant baseline score. This suggests that each of these can contribute to the disambiguation of ambiguous terms in biomedical text. In addition, disambiguation is improved by combining information sources. This is consistent with results over general text. For example, Stevenson and Wilks [10] and Harley and Glennon [28] showed that WSD could benefit from use of several different types of information from a dictionary. More recently Specia *et al. *[11] showed that a combination of information sources could improve disambiguation of Portuguese verbs.

Combining MeSH terms with other features generally improves performance, suggesting that this provides the classifier with information not available from the others. An important difference between MeSH terms and the other features (linguistic and CUIs) is that they are assigned to the entire abstract rather than just individual terms and, as such, provide information about the topic of the abstract which would be hard to derive from more local features. This can be seen in the example usage of "adjustment" in the Features section above. The abstract in which this term is used discusses the treatment of coronary angioplasty using heparin, an anticoagulant. This abstract does not include the term "anticoagulant" but is assigned the MeSH term "D27.505.954.502.119: Anticoagulants". It would be difficult to determine that this abstract discusses anticoagulants using only the kinds of linguistic features used by many WSD systems. However, MeSH terms provide a way of identifying this information. These findings in this study are consistent with results from WSD of general text. For example, Agirre and Martinez [21] observed a small improvement when domain information was used as additional information in their WSD system.

Unlike MeSH terms, the inclusion of CUIs as features does not always improve performance and, in several cases, causes it to fall. This is consistent with McInnes *et al. *[17] who concluded that CUIs were a useful information source for disambiguation of biomedical text but that they were not as robust as one type of linguistic information (unigrams) which they had used for a previous system. However, in some ways this result is surprising since CUIs are derived from UMLS, a resource which contains all the information in the MeSH hierarchy (the MeSH hierarchy is a subset of UMLS). The most likely reason for this is that our CUI assignment, provided by MetaMap, is automatic. MetaMap does not attempt to disambiguate terms which map onto more than one UMLS concept so this CUI assignment is noisy.

Differences between CUIs and MeSH terms were explored further through an analysis of their distribution in the NLM-WSD corpus. A first observation is that CUIs are far more frequent than MeSH terms. On average 489 CUIs are assigned to each abstract in the NLM-WSD data set and only 13.8 MeSH terms. Two measures were used to determine how well CUIs and MeSH terms indicate the meaning of an ambiguous term. The first of these, entropy, is a measure of uncertainly [29]. Lower entropy values indicate there is less variation in the meanings of abstracts to which a particular CUI or MeSH term is assigned. Entropy is computed using equation 1 where *F *is a feature (such as CUI or MeSH term), *n *is the total number of senses and *p*_*i *_is the probability that an instance of that feature is assigned to an abstract which has been assigned a particular sense (*i*).

(1) $$Entropy(F)=\sum_{i=1}^{n}-p_{i}\;log_{2}p_{i}$$

The entropy of each feature (CUI or MeSH term) is computed and averaged across all terms in the data set. For CUIs this figure is 0.389, significantly higher than the equivalent figure for MeSH terms, 0.275 (Wilcoxon Signed Ranks Test, *p *< 0.01). The higher entropy figure indicates that CUIs provide less information about the sense of an ambiguous term than MeSH terms.

An additional metric, Information Gain [30], is based on entropy and provides a measure of how useful a feature is to classify the data. It can be used to determine how accurately a CUI or MeSH term indicates the sense being used in an abstract to which it has been assigned. Information Gain is computed using the formula shown in equation 2 where *C *is a collection of texts and *V alues*(*F*) the set of values which the feature *F *can be assigned. In our case the collection, *C*, is the set of abstracts for a given term in the NLM-WSD collection and *V alues*(*F*) is binary, since each CUI and MeSH term is either assigned to a particular abstract or not.

(2) $$Information\;Gain(C,F)=Entrophy(C)-\sum_{v\in Values(F)}\frac{\left|C_{v}\right|}{\left|C\right|}Entropy(C_{v})$$

The average Information Gain score for all features which apply to a term was computed. The average of this figure across all terms is 0.014 for CUIs and significantly higher (0.017) for MeSH terms (Wilcoxon Signed Ranks Test, *p *< 0.01). This indicates that MeSH terms provide more useful information for sense classification than CUIs.

The methodology adopted in this study has been to evaluate and compare a variety of types of information which may be useful for the disambiguation of biomedical terms. Each of these sources are readily available: linguistic features can be extracted directly from text, MeSH terms are available for many MEDLINE entries while CUIs can be generated by MetaMap. The study does not directly compare the usefulness or value of the MeSH hierarchy against UMLS since we are using manually assigned MeSH terms and CUIs from UMLS which are automatically generated. We do not have access to a reliable assignment of CUIs to text; if we had WSD would not be necessary since the senses used in the NLM-WSD corpus are effectively CUIs. However, our study suggests that using linguistic features is a better strategy for WSD of biomedical terms, confirming previous results [17]; in addition, there is nothing to be gained from combining CUIs with linguistic features. On the other hand, our study also shows that MeSH terms can improve disambiguation performance and should be used if available (such as disambiguation of terms in MEDLINE abstracts). While the benefit provided by MeSH terms is statistically significant, it is quite small and not crucial for disambiguation of biomedical text.

## Conclusion

This paper has compared a variety of information sources for WSD of ambiguous biomedical terms and reported results which exceed the performance of previously published approaches. We found that the most accurate results can be achieved using a combination of linguistic features commonly used for WSD of general text and manually assigned MeSH terms. While CUIs are a useful source of information for disambiguation, they do not improve the performance of the best system configuration, i.e. when used in addition to linguistic features and MeSH terms. This may be because our approach uses manually assigned MeSH terms while the CUIs are obtained automatically using MetaMap. Analysis of the information gain afforded by automatically assigned CUIs versus manually assigned MeSH terms for the sense classification task confirms that the MeSH terms do indeed supply more information.

The linguistic information used in this paper comprises a wide variety of features including unigrams, local collocational features and salient bigrams. When these feature types are considered singly unigrams are the most effective, while unigrams together with local collocations are the most effective pair. We have not explored the contribution of individual collocational features, however, and this is a topic for further work. In addition, our approach does not make use of the fact that MeSH terms are organised into a hierarchy. It would be interesting to discover whether this information could be used to improve WSD performance.

Others, for example [31], have developed techniques to make use of hierarchical information in WordNet for WSD which could be adapted to MeSH.

## Competing interests

The authors declare that they have no competing interests.

## Authors' contributions

DM and YG developed the WSD system used in this study. YG generated all experimental results. MS carried out analysis of previous approaches and, together with RG, conceived the study and co-ordinated its design and the drafting of this manuscript. All authors read and approved the final manuscript.
